# Interplay of Stem Cell Characteristics, EMT, and Microtentacles in Circulating Breast Tumor Cells

**DOI:** 10.3390/cancers5041545

**Published:** 2013-11-14

**Authors:** Monica Charpentier, Stuart Martin

**Affiliations:** 1 Program in Molecular Medicine, University of Maryland School of Medicine, 655 W. Baltimore St., Bressler Bldg., Rm 10-20, Baltimore, MD 21201, USA; E-Mail: monica.charpentier@som.umaryland.edu; 2 Marlene and Stewart Greenebaum National Cancer Institute Cancer Center, University of Maryland School of Medicine, 655 W. Baltimore St., Bressler Bldg., Rm 10-29, Baltimore, MD 21201, USA; 3 Department of Physiology, University of Maryland School of Medicine, 655 W. Baltimore St., Bressler Bldg., Rm 10-29, Baltimore, MD 21201, USA

**Keywords:** circulating tumor cells, breast cancer, cancer stem cells, EMT, microtentacles, detyrosinated tubulin

## Abstract

Metastasis, not the primary tumor, is responsible for the majority of breast cancer-related deaths. Emerging evidence indicates that breast cancer stem cells (CSCs) and the epithelial-to-mesenchymal transition (EMT) cooperate to produce circulating tumor cells (CTCs) that are highly competent for metastasis. CTCs with both CSC and EMT characteristics have recently been identified in the bloodstream of patients with metastatic disease. Breast CSCs have elevated tumorigenicity required for metastatic outgrowth, while EMT may promote CSC character and endows breast cancer cells with enhanced invasive and migratory potential. Both CSCs and EMT are associated with a more flexible cytoskeleton and with anoikis-resistance, which help breast carcinoma cells survive in circulation. Suspended breast carcinoma cells produce tubulin-based extensions of the plasma membrane, termed microtentacles (McTNs), which aid in reattachment. CSC and EMT-associated upregulation of intermediate filament vimentin and increased detyrosination of α-tubulin promote the formation of McTNs. The combined advantages of CSCs and EMT and their associated cytoskeletal alterations increase metastatic efficiency, but understanding the biology of these CTCs also presents new therapeutic targets to reduce metastasis.

## 1. Introduction

### 1.1. Breast Cancer Metastasis

In the United States, improved early detection and new treatments have led to a decline in the overall mortality due to breast cancer, but the survival rates for patients with metastatic disease have not improved significantly [1]. As a complex and heterogeneous disease, there currently is no standardized or curative therapy for metastatic breast cancer [2]. With metastasis responsible for the vast majority of breast cancer-related deaths, research into the mechanisms of breast cancer metastasis warrants urgent attention.

Metastasis is a multi-step process whereby cancer cells face numerous challenges. They must: (1) detach from the primary tumor, (2) invade through the basement membrane, (3) enter the bloodstream or lymphatic system, (4) resist anoikis (detachment-induced apoptosis), (5) evade immune surveillance, (6) exit circulation, (7) persist and (8) eventually colonize a distal site [3]. Metastasis was traditionally thought of as the final step in the linear progression of breast cancer, developing long after primary tumor formation, as multiple stepwise genetic mutations would need to accumulate before carcinoma cells could acquire the ability to migrate from the primary tumor and enter circulation [4]. However, recent evidence suggests that metastasis may be a much earlier event than previously suspected, where cancer cells competent for metastasis can disseminate early during primary tumor development [4]. In a HER2 PyMT mouse model and a PyMT mouse model, disseminated breast cancer cells could be detected in the bone marrow and the lungs, respectively, even before the full development of a primary tumor [5,6]. The development of single cell whole genome analysis allowed researchers to extend these studies to patients, where they found early disseminated breast cancer cells with fewer genetic changes than the cells from the primary tumors, suggesting that breast cancer cells can metastasize very early in tumor development, leading to the observed differences in genetic mutations between the primary tumors and the metastatic lesions [7].

### 1.2. Animal Models of Metastasis

Animal models of metastasis are critical tools for improving our understanding of the biology of metastasis and for developing pharmaceutical compounds that can prevent or reduce metastatic spread. Murine modeling remains the mainstay for studying cancer metastasis. Multiple animal models for mimicking human cancer metastasis exist, with varying degrees of success in recapitulating the features of human metastasis. There are three general classes of mouse models of human cancer cell metastasis: (1) ectopic modeling implanting human tumor cells subcutaneously in nude mice; (2) orthotopic modeling implanting human tumor cells into the murine equivalent organ of their primary site; and (3) experimental metastasis models directly injecting human cancer cells into the murine circulation, either via the tail-vein or by intracardiac injection (reviewed in [8,9]).

Importantly, mouse modeling can also be used to study the CTC phase of metastasis, where cancer cells are suspended in the circulatory system. Technology for imaging and modeling CTCs in mice has rapidly developed. The use of human cancer lines labeled with fluorescent proteins such as GFP provides a powerful tool for non-invasive imaging to study the real-time behavior of cancer cells during the process of metastasis (reviewed in [10,11]).

Using green fluorescent protein (GFP)-labeled PC-3 prostate cancer cells, Glinskii *et al.* demonstrated that orthotopic xenografts generated viable CTCs that could be expanded after FACS sorting for GFP-positive cells from cardiac puncture [12]. This orthotopic model of PC-3-GFP cells produced extensive metastases and a high load of CTCs, compared to ectopic modeling of PC-3-GFP cells, which rarely generated metastases and were unable to generate viable CTCs [12]. Importantly, these GFP-PC-3 cells isolated from circulation were highly metastatic when reimplanted orthotopically [12]. Taking advantage of FACS analysis, they next orthotopically coimplanted the GFP-PC-3 cells isolated from circulation alongside an equivalent number of parental RFP-PC-3 cells. These coimplantation studies revealed a vastly greater enrichment of the CTC-derived PC-3-GFP cells compared to the parental RFP-PC-3 cells in circulation as well as in bone marrow and lymph node metastatic lesions. These results suggest that CTCs recovered from orthotopically injected metastasis models are highly capable of metastasis, potentially due to their successful survival under the extreme selective pressures imposed in the circulation [12]. Importantly, prostate cells derived from circulation after orthotopic implantation in a prostate mouse metastasis model had increased anoikis resistance and survival in suspension compared to parental cells that had not been selected from circulation [13]. These CTC-derived cells had increased mRNA and protein expression of inhibitor of apoptosis proteins (IAPs) [13]. Transfection of XIAP into anoikis-sensitive parental cells promoted anoikis resistance, while siRNA silencing of XIAP in CTC-derived cells lead to greater anoikis sensitivity [13]. Notably, small molecule inhibitors of XIAP selectively reduced viability in suspended cells and selectively reduced metastatic spread, rather than primary tumor growth, in an orthotopic mouse model [13].

Similar to methods used to detect CTCs in human patients, immunomagnetic beads can capture CTCs in mouse models of CTCs from orthotopic injection of GFP-PC-3 cells, confirmed by fluorescence microscopy [14]. This method of using immunomagnetic beads to capture fluorescently-labeled, viable CTCs in an orthotopic metastasis model provides a method to study the factors enabling successful metastasis *in vivo*, which is particularly useful because the captured cells readily proliferate for further biological studies. Similarly, the CellTracks System (Janssen Diagnostics, LCC, Raritan, NJ, USA) was adapted for use in enumerating and fluorescently-analyzing CTCs from breast cancer xenografts, although this system requires a fixation step that precludes further functional study [15].

### 1.3. Patient CTC Detection

Up to 40% of patients with occult metastases detected in the bone marrow had no clinical symptoms or signs of metastatic disease by imaging [16]. To improve outcomes for patients with metastatic disease, we will need the ability to detect cancer cells in the process of metastasis at much earlier time points. The development of new technologies now allows the detection of circulating tumor cells (CTCs) in the bloodstream of patients with primary or advanced disease. A multicenter, prospective trial of patients with metastatic breast cancer using the CellSearch System (Janssen Diagnostics, LCC, Raritan, NJ, USA) found that the presence of CTCs was a robust independent prognostic indicator of progression-free survival and overall survival [17,18]. The CellSearch system is currently the only US Food and Drug Administration-approved test for using CTC enumeration in a clinical setting [19]. CTC enumeration is an earlier and more reproducible method for assessing disease progression than radiological imaging, and can even be used to predict survival in patients with non-measureable metastatic breast cancer [20,21]. As methods to isolate and characterize CTCs develop, enumeration and characterization of CTCs from peripheral blood samples will be able to provide prognostic and predictive information throughout the course of a breast cancer patient’s treatment in a relatively non-invasive manner. In addition to its utility in clinical decision-making, this emerging technology to isolate and characterize circulating tumor cells is opening the door to directly test two intersecting theories that are changing the paradigms of breast cancer progression: the cancer stem cell hypothesis and epithelial-to-mesenchymal transition.

## 2. The Cancer Stem Cell Hypothesis

### 2.1. Mammary Stem Cells

Somatic stem cells serve to populate the tissues of the body but are generally constrained by their ability to differentiate only into organ-specific cell types. Parallels have been drawn between the characteristics of these tissue-specific stem cells and tumor cells, leading to the cancer stem cell (CSC) hypothesis that tissue stem cells are the cell of origin for tumor development and the target for oncogenic transformation [22,23]. Tissue stem cells have long lifespans in order to repopulate organs as needed throughout the organism’s lifetime, rendering them more susceptible to the accumulation of oncogenic mutations than short-lived mature tissue cells; thus tissue stem cells are a more likely cell of origin for tumor development [24]. The mammary gland has the potential to remodel throughout adult life, therefore requiring a persistent population of cells with the stem cell characteristics of self-renewal and differentiation. The mammary gland undergoes significant self-renewal during each pregnancy cycle, as evidenced by the proliferation and differentiation which occur during pregnancy to increase the population of alveolar cells required for milk production and secretion, and the massive apoptosis which occurs as lactation ends and the gland returns to a pre-pregnant state. This cycle of tissue regeneration may repeat for multiple pregnancies, requiring breast stem cells to repopulate the mammary gland [24].

The identification of surface markers to enrich for mouse mammary stem cells paved the way for studies supporting the existence of these mammary stem cells. Using the surface markers Lin^−^/CD29^hi^/CD24^+^ to enrich for murine mammary stem cells, a single isolated cell can reconstitute an entire functional mammary gland, providing convincing evidence for the existence of mammary stem cells [25,26]. These murine mammary stem cells are also capable of self-renewal, as demonstrated by serial transplantation assays [25]. Stem cells may divide asymmetrically to produce one daughter stem cell and one progenitor cell, which then differentiates into the different tissues lineages, or stem cells may divide asymmetrically, producing two identical stem cells and thus expand the proportion of stem cells in the tissue [27]. Asymmetric stem cell division leading to an increase in the proportion of cancer stem cells may account for the variation in the proportion of stem cells seen in the literature. 

### 2.2. Breast Cancer Stem Cells

Subversion of the normal stem cell signaling pathways by a mutated tissue stem-cell may drive both dysregulated proliferation, leading to tumorigenicity, and aberrant differentiation, leading to tumor heterogeneity. Traditional models suggesting that any of the cancer cells in a heterogeneous tumor may be continuously replicating and are equally capable of recreating a tumor or metastasis are not supported by studies showing that many thousands of these cancer cells must be transplanted into mice in order to generate tumors [28]. In contrast, the cancer stem cell hypothesis suggests that while the tumor is a heterogeneous population of cells, only the cancer stem cells are continuously dividing and repopulating the tumor [22]. In the cancer stem cell hypothesis, tumor heterogeneity arises from the division of a cancer stem or progenitor cell into lineage-specific cells. 

The identification of CSCs on the basis of unique surface marker phenotypes began in leukemia and brain cancers, and this technique for identifying CSCs continued in the hunt for breast CSCs. Based on the premise that only the CSC subpopulation is tumorigenic, studies beginning with the well-validated leukemia CSC model frequently use the number of cells needed to form a tumor in mice as an indication of the proportion of stem cells in a population, with populations that form tumors from fewer cells more representative of a stem cell population [29,30]. Flow cytometry was used to separate human primary and metastatic breast cancer cells on the basis of cell surface markers, showing that a CD44^hi^/CD24^lo^ and lineage-marker-absent population of cells from breast cancer patients, but not CD44^lo^/CD24^hi^ cells, was enriched in the ability to form tumors in NOD/SCID mice [28]. As few as 100 CD44^hi^/CD24^lo^/lin^−^ cells could form tumors in mice, while tens of thousands of unenriched cells failed to form tumors [28]. The composition of these tumors recapitulated the heterogeneity of the original tumor, suggesting the existence of breast CSCs that are not only capable of self-renewal leading to the production of a tumor, but also capable of generating cells that are able to differentiate and constitute the bulk of the heterogeneous tumor. Thus the selected cancer cells displayed the identifying characteristics of tissue stem cells [28]. These tumors continued to contain a CD44^hi^/CD24^lo^/lin^−^ subpopulation of cells, which remained tumorigenic when transplanted again into mice a second time while the unsorted tumor bulk cells were not tumorigenic, providing support for the existence of breast CSCs that are able to divide to produce both stem cells capable of further division and differentiated progeny that lack such capability.

Functional assays for stem cell character are important for validating the use of phenotypic surface markers. Based upon the model in which stem cells, including cancer stem cells, have innate chemotherapeutic resistance, a functional assay was developed initially in hematopoietic cells to select for CSCs based upon the expression of one mechanism of chemotherapeutic resistance, aldehyde dehydrogenase [31,32,33,34]. Aldehyde dehydrogenase ALDH1 was shown in hematopoietic cells to be the only ALDH enzyme critical for the regulation of stem cell activity. ALDH1 is present in cells from human breast tissue that have phenotypic and functional stem cell character and thus detection of ALDH1 expression via the ALDEFLUOR reagent has been used to isolate a putative cancer stem cell population from human breast carcinomas [35]. This population of putative breast CSCs can often overlap to a small degree with the CD44^hi^/CD24^lo^/lin^−^ population; however, most cell lines and tumors possess their own unique ratio of stem cell markers and populations. The observation that different sets of markers identify distinct and separate populations of self-renewing cells demonstrates a limitation in the ability of surface markers to accurately and consistently define CSC populations. Thus functional determinants of the CSC population, such as the ability to recapitulate heterogeneous tumors similar to the phenotype of the original tumor upon limiting dilutions in mice, remain the most persuasive evidence for the existence of breast CSCs.

Attempts to study mammary progenitor cells *in vitro* have been hampered by the inability to grow progenitor cells in large enough quantity for experimentation without inducing differentiation because traditional, adherent cell culture conditions promote differentiation of progenitor cells. Pioneering work by Dontu *et al.* is significant because it marks the first development of an *in vitro* system for the continuous propagation of non-adherent human mammary epithelial cells without differentiation [36]. The mammosphere assay is based on the culture of mammary epithelial cells under non-adherent conditions, as normal mammary epithelial cells are unable to survive without attachment to a substrate and die by anoikis, while stem cells survive and are enriched in floating spheres [37]. In addition to serving as a system for the continuous culture of mammary stem cells in an undifferentiated state, the mammosphere culture system can also serve as an assay for the indirect measurement of stem cell character, because mammosphere formation depends on the presence of self-renewing stem cells [36]. Further studies have confirmed that mammospheres contain stem-like cells that can generate an entire mammary ductal tree when implanted into a cleared mouse mammary fat pad [38].

While evidence has been accumulating in support of the CSC hypothesis in breast carcinogenesis, it is far from becoming widely accepted. One troubling aspect of the cancer stem cell theory is that it is unclear whether or not the cancer stem cell population is indeed a very small subset of the tumor population, as some studies in lymphoma and melanoma have shown that a much larger than expected proportion of the population is tumorigenic [39,40]. This melanoma study illustrates the limitations of our *in vitro* and *in vivo* models for cancer stem cells, as it used the NOG mouse as a model host, which is further immunocompromised than the NOD/SCID mice used in most of the preceding CSC studies, and thus more easily accepts heterologous cells. The increased tumorigenicity seen using the NOG mouse model suggests that the host immune system interacts in crucial but yet undefined ways with the cancer stem cell population. Indeed, a recent study has shown the potential for immune targeting of breast CSCs [41]. Additionally, it is unclear whether breast CSCs originate by the oncogenic transformation of normal mammary stem cells or by the dedifferentiation and acquisition of stem-cell characteristics by carcinoma cells, or if both pathways contribute to the generation of breast CSCs. Recent studies indicate that reprogramming non-tumorigenic mammary epithelial cells with embryonic stem cell transcription factors OCT4, SOX2, Klf-4 and c-Myc can generate breast CSCs [42]. While further research is needed to definitively address the origin of breast CSCs, the CSC theory itself has become well-established and highlights the need to target breast CSCs if we hope to prevent or reduce breast cancer mortality.

## 3. CSCs and Metastasis

Most research has focused on the role of breast CSCs in primary tumor development, but recent studies suggest that breast CSCs may also play a critical role in metastasis. If the cancer stem cells are the only cells in a tumor with inherent tumorigenicity, then it is reasonable to conclude that the cancer stem cells are involved in the metastatic process, as metastatic outgrowth requires many of the same characteristics as primary tumor development [43]. Emerging evidence from mouse models of metastasis and from studies of human breast cancer patients suggest that CSCs may be the critical cells responsible for metastasis. ALDEFLUOR was used to identify cancer stem cell-like populations of breast cancer cell lines, which demonstrated *in vivo* stem cell activity by tumorigenicity assays in mice. Importantly, these breast CSCs were shown to have increased metastatic potential by intracardiac injection into NOD/SCID mice and subsequent luciferase assays for metastasis formation [44]. In support of these findings, additional studies show that the breast cancer stem cell population defined by the CD44^hi^/CD24^lo^ population may be more associated with metastasis than primary tumor development [45]. A recent study used an optical reporter fusion gene to track as few as 10 CD44^hi^/lin^−^ human patient tumor cells in a mouse xenograft model, where they saw an enrichment of breast cancer stem cells in spontaneous metastases, suggesting that breast CSCs are more capable of metastasis [46]. Importantly, studies in human breast cancer patients also suggest that breast CSCs are essential to metastasis. Disseminated tumor cells in human breast cancer patients were recently shown to express the stem cell phenotype CD44^hi^/CD24^lo^ [47]. Additionally, another study showed that 70% of patient blood samples that were positive for circulating tumor cells that expressed the functional stem cell marker ALDH1 [48]. Interestingly, patients with triple-negative breast cancer have a poor prognosis; relapsed tumors and distant metastases of triple-negative breast cancers are associated with CSCs [49]. These findings suggest that an increase in CSCs highly competent for metastasis may be responsible for the worse prognosis of triple-negative breast cancer. While this research provides strong evidence linking breast CSCs with metastasis, very challenging studies to track a single cell with CSC characteristics through circulation and development into a metastatic lesion will be required to definitively demonstrate that breast CSCs are the cells responsible for metastasis. The role of breast CSCs in metastasis may be distinct from their role in primary tumor carcinogenesis, as successful metastasis requires significant changes to cellular morphology and signaling pathways as the cancer cells travel through and respond to the different microenvironments en route to a site of dissemination.

## 4. Cytoskeletal Alterations as Breast Cancer Cells Enter Circulation

### 4.1. EMT and Circulating Tumor Cells

Dynamic cytoskeletal alterations are one of the most critical requirements for metastasis, as breast cancer cells must dissociate from the primary tumor site, migrate towards the bloodstream, and intravasate to become circulating tumor cells (CTCs). The epithelial-to-mesenchymal transition (EMT) has been implicated as a mechanism responsible for endowing epithelial cancer cells with the necessary traits and cytoskeletal alterations for successful metastasis. EMT is well characterized as an embryonic process whereby epithelial-like cells in the developing embryo convert into motile mesenchymal cells that can migrate to alternative sites in the embryo. This developmental program is thought to occur in a deregulated manner in epithelial cancers, allowing stationary, polarized breast cells tightly connected to adjacent cells to disband their cell-cell junctions and invade as non-polarized single cells [50,51,52]. Activation of the EMT program leads to downregulation of epithelial proteins required for maintenance of the polarized epithelial sheet such as occludins, E-cadherin, and claudins, and upregulation of more plastic mesenchymal proteins such as vimentin, N-cadherin, and smooth muscle actin.

The majority of the studies of EMT’s effects on the cytoskeleton focused on cancer cells attached to the extracellular matrix (ECM), which is a dramatically different environment than a CTC in circulation. This research on attached cells heavily stresses the role of actin reorganization and actin-based motility structures. How the EMT program may help cancer cells resist anoikis and survive in circulation as CTCs is a currently developing area of research. In the bloodstream, the mechanical forces on a CTC are vastly different from those when the cell is migrating or invading within tissues. In circulation, epithelial cells tend to die from anoikis, an apoptotic cell death that occurs in response to detachment. Prior activation of the EMT program during the initial invasion steps of metastasis may provide resistance to anoikis once in the bloodstream. EMT-induced loss of cell polarity in metastatic cancer cells can help downregulate the Hippo pathway, leading to resistance to anoikis [53,54]. One of the hallmarks of EMT, loss of E-cadherin, also serves as a mechanism for anoikis resistance. Loss of E-cadherin at the cell membrane disrupts a complex including Ankyrin-G and NRAGE that renders cells sensitive to apoptosis; when this complex is lost, NRAGE translocates to the nucleus, where it can form a repressor complex to prevent expression of the p14ARF gene, an anoikis sensitizer, to confer anoikis resistance [55]. EMT transcription factors may also contribute to anoikis resistance, as master EMT regulator Twist prevents expression of p14ARF, thus conferring anoikis resistance [56]. 

Many studies suggest that EMT occurring in metastasizing cancer cells is induced by microenvironmental factors such as TGF-β, leading to reversible changes in master regulators of the EMT program, such as transcription factors Twist and Snail, or epigenetic modulation by changes in microRNA expression of the mir200 family, rather than by permanent genetic changes [50,51]. The complex reprogramming of both signaling pathways and cytoskeletal reorganization during an EMT may necessarily be a transient phenomenon, as the cancer cells must revert back to a more epithelial state in order to eventually grow out as metastatic lesion. A recent study using Twist regulation to control epithelial or mesenchymal status revealed that a reversible or transient EMT is required for disseminated mesenchymal cancer cells to proliferate and colonize a distant site for metastatic outgrowth [57].

### 4.2. EMT-Directed Cytoskeletal Alterations May also Promote the Metastatic Potential of CTCs

In addition to aiding in detachment from the primary tumor, invasion, and anoikis resistance in circulation, EMT also has specific effects on the cytoskeleton of breast cancer cells that affect the tumor cell during the CTC phase of metastasis. The environment of the circulatory system presents a unique challenge for circulating epithelial tumor cells, and they must develop a more flexible cytoskeleton to avoid fragmenting in small capillary beds due to shear forces in circulation [58]. The cellular tensegrity model describes the balance of cytoskeletal forces on a suspended epithelial cell: the inward-directed force of the actin cortex is balanced with an outward-directed microtubule force to maintain cellular morphology [59]. Existing in a state of ECM detachment, a CTC no longer has connection to the ECM to assist in generating traction; indeed, a less rigid cellular shape is essential for a CTC to deform and survive passage through narrow capillaries [58]. Interestingly, this more deformable CTC may be a characteristic associated both with transformation and with stemness [60,61].

**Figure 1:**
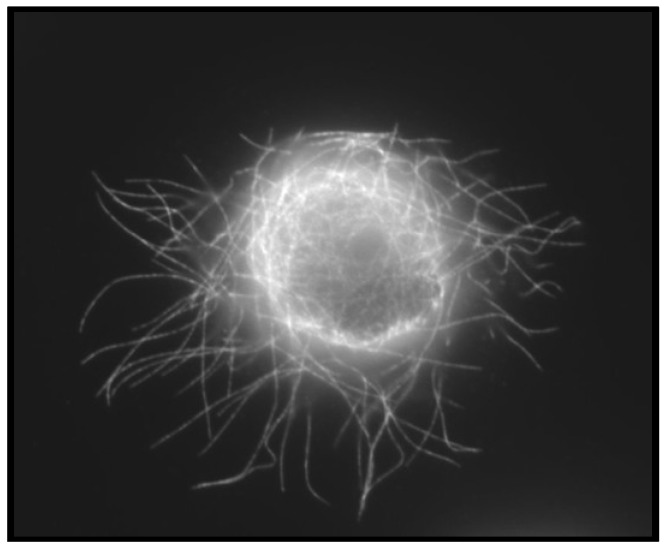
Breast cancer cells produce microtentacles in response to detachment. Confocal microscopy of a suspended breast tumor cell reveals tubulin-based long, flexible microtentacles.

After surviving in circulation, a CTC must first attach to the capillary endothelium and then extravasate into the tissue to effectively develop into a metastatic lesion. The mechanism by which CTCs reattach to the capillary endothelium is poorly characterized. Intravital imaging studies using colon carcinoma cells revealed that CTCs attach to the liver sinusoid capillaries in a microtubule-dependent, but not actin-dependent manner [62]. Recent studies modeling CTCs using suspended breast carcinoma cells have identified a novel cellular structure, termed microtentacles (McTNs, seen in Figure 1), which may explain these findings. McTNs are dynamic microtubule-based protrusions extending from the plasma membrane of carcinoma cells detached from the extracellular matrix [63]. Tubulin-based, McTNs are mechanistically distinct from actin-based structures such as invadopodia and filopodia, and are antagonized by the inwardly-directed actin cortex, matching the mechanism of the *in vivo* studies of CTC retention [62,63,64]. Functionally, McTNs promote the reattachment of suspended breast carcinoma cells, a critical step in metastasis necessary for CTCs to exit the bloodstream [63,65,66]. Live confocal imaging revealed that flexible McTNs on attaching tumor cells penetrate the junctions between endothelial cells [67]. Importantly, McTNs are increased in frequency and length in breast cancer cell lines with increasing metastatic potential, and persist on the surface of suspended, anoikis-resistant breast epithelial cells for several days [63,66]. These data support a model that McTN formation in response to carcinoma cell detachment could enhance the reattachment and metastatic potential of CTCs.

McTNs are promoted by specific cytoskeletal modifications. Matching the mechanism of *in vivo* CTC reattachment, inhibitors of actin polymerization increase the formation of McTNs, while inhibitors of tubulin polymerization decrease McTNs [62,63]. Microtubules are composed of α- and β-tubulin heterodimers, and can be regulated by multiple post-translational modifications [68,69]. The carboxy-terminal tyrosine on α-tubulin is subjected to cyclical removal, by a yet-unidentified tubulin carboxypeptidase (TCP), to generate detyrosinated tubulin (Glu-tubulin) and re-ligation, by tubulin tyrosine ligase (TTL), to regenerate tyrosinated tubulin (Tyr-tubulin). Glu-tubulin microtubules have a vastly increased stability *in vivo*, persisting for hours rather than the 3 to 5 minutes seen in microtubules composed of Tyr-tubulin [70]. Interestingly, protein levels of Glu-tubulin increase dramatically immediately upon detachment and are found enriched in the McTN protrusions rather than the cell body [63]. Importantly, tubulin detyrosination in breast cancer predicts poor prognosis and metastasis [71,72]. In an immunohistochemistry study of 134 breast cancer patient samples, tubulin detyrosination was associated with tumor aggressiveness, and when combined with the Scarf-Bloom-Richardson grade, significantly correlated with poor clinical outcome [72]. In a later immunohistological study comparing 78 cases of malignant breast cancer and 69 benign cases, Glu-tubulin staining was observed in 71.8% of malignant cases but only 8.3% of benign tumor samples and 4.7% of samples of other benign breast disease, providing a significant association between the presence of Glu-tubulin and malignancy [71].

Microtubules can increase their stability by associating with intermediate filaments, an association that also enhances McTN formation. Western blotting and immunofluorescence of suspended breast carcinoma cells revealed that vimentin intermediate filaments, but not cytokeratins, extend into McTNs and that vimentin-expressing breast cancer cell lines had higher McTN frequencies [66]. These data match studies using cell lines generated from micrometastatic lesions in the bone marrow of breast cancer patients revealing a downregulation of cytokeratins and an upregulation of vimentin [73]. Furthermore, disrupting vimentin through phosphatase PP1/PP2A inhibition or by dominant-negative vimentin expression reduced McTN extension and inhibited reattachment from suspension [66]. Vimentin has been previously shown to preferentially interact with detyrosinated tubulin and, importantly, vimentin coaligns with Glu-tubulin in McTNs to enhance McTN formation [66,70,74].

The cytoskeletal alterations promoting McTN formation, increased vimentin expression and increased tubulin detyrosination, are enhanced in EMT. Vimentin is an intermediate filament strongly associated with EMT, and is a poor prognostic indicator in triple negative breast cancer [75,76]. Induction of an EMT through ectopic Twist or Snail expression increased detyrosinated tubulin levels and enhanced McTN formation and subsequent reattachment from suspension by downregulating tubulin tyrosine ligase (TTL) expression [67]. Importantly, clinical tumor samples display a concordant elevation of Twist and Glu-tubulin expression at the invasive front of patients with ductal carcinoma *in situ*, suggesting that EMT can promote microtubule stability as breast cancer cells escape the tissue and become CTCs [67]. Since EMT and vimentin are also induced at epithelial wound edges, this raises the possibility that migration from the primary tumor could expose tumor cells to wounding stimuli that would only increase when CTCs enter the free-floating environment of the circulation [77,78]. 

### 4.3. Convergence of EMT and CSCs

The CSC hypothesis and EMT both provide compelling insight into the mechanisms of metastasis; recent studies revealing the convergence of these two research fields has considerable ramifications for our understanding of breast cancer progression. When human mammary epithelial cells undergo a forced EMT by the expression of transcription factors Twist or Snail, they acquire the CD44^hi^/CD24^lo^ breast cancer stem cell phenotype and increased mammosphere formation, a measure of stem cell self-renewal [79]. Importantly, inducing EMT in tumorigenic mammary epithelial cells dramatically increased their tumorigenicity in immunocompromised mice, the gold standard for identifying cancer stem cells [79]. In complementary experiments, naturally-arising normal and neoplastic human mammary epithelial stem-like cells displayed mesenchymal morphology and a gene expression pattern associated with EMT [79]. The precise molecular mechanisms connecting EMT and CSCs are just beginning to be appreciated. One of the EMT transcription factors used in the landmark study connecting EMT and CSCs, Twist, may itself increase self-renewal by directly stimulating CSC polycomb complex protein Bmi1 and by downregulating the CSC phenotypic surface marker CD24 [80,81]. Epigenetic regulation by the mir200 family has been implicated in maintaining both an EMT and a CSC phenotype, as well as regulating cell motility and anoikis resistance [82]. Importantly for CTCs and metastasis, both EMT and CSCs may lead to a more deformable cytoskeleton, a requirement for successful transit through small capillaries [60]. This discovery that EMT is linked to the acquisition of CSC traits suggests a dangerous combination in which may prime CTCs for more successful metastasis. Not only would these breast cancer cells have the EMT-like ability to invade, migrate, and become CTCs, but with the CSC traits of enhanced tumor-initiation, self-renewal, and chemotherapeutic resistance, these CTCs are primed for more successful metastatic outgrowth.

## 5. Emerging Evidence for EMT, CSC, and Related Cytoskeletal Alterations in CTCs from Breast Cancer Patients

While EMT and CSCs have been implicated in the metastatic process, definitive evidence in cancer patients has been limited by available technology to observe CTCs during the process of metastasis. Recent technology to isolate CTCs from the peripheral blood of breast cancer patients has created a unique opportunity to assess CTCs during the metastatic process. Multiple studies of metastatic breast cancer patients using the CellSearch System (Janssen Diagnostics, LCC, Raritan, NJ, USA) have found that the presence of CTCs was a robust independent prognostic marker of progression-free survival and overall survival [17,83]. CTC enumeration methods like CellSearch and other studies on breast cancer patient CTCs and disseminated tumor cells have generally relied on immunohistochemistry or flow cytometry to determine if such cells in the process of metastasis express markers implicating EMT or CSCs. In a recent prospective study of patients with early breast cancer, the presence of disseminated CD44^hi^/CD24^lo^ CSCs in the bone marrow was identified for the first time as an independent predictor for reduced disease-free survival [84]. Differential centrifugation, magnetic cell separation, immunostaining, and flow cytometry were used to extend these studies by directly identifying CTCs with the CD44^hi^/CD24^lo^ CSC phenotype from the peripheral bloodstream, providing further evidence that CSCs are important in breast cancer metastasis [85,86,87]. CSCs have previously been associated with poor prognosis in triple negative breast cancer in immunohistochemistry studies on relapsed tumors and metastases, and patients with triple negative primary breast cancers were more likely to have CD44^hi^/CD24^lo^ cells disseminated in the bone marrow [49,88,89]. These studies have used recently-developed technology to detect CTCs and disseminated cancer cells with breast CSC characteristics. To determine if these rare stem-like CTCs are responsible for later metastasis, our CTC technologies need to be further refined to rapidly and reproducibly isolate live and intact CTCs for functional studies of metastasis. CTC technology is rapidly evolving, and will soon allow for such testing of metastatic competence. Already, a study examined the metastatic competence of patient-derived CTCs *in vivo* by expanding the CTCs in culture and injecting them into the tail-vein or intracardiacally into immunodeficient mice to find metastases to the brain and lung [90]. A recent study injected CTCs from patients with progressive metastatic breast cancer into immunocompromised mice to demonstrate the existence of metastasis-initiating cells (MIC) that could give rise to bone, lung, and liver metastases [91]. The metastatic efficiency of the bulk isolated CTCs was low, but the MIC cells were found to express EpCAM, CD44, CD47, and MET [91]. The levels of CD44^+^/MET^+^/CD47^+^ CTCs paralleled clinical progression and was a better indicator of overall survival than the frequency of bulk CTCs [91].

**Figure 2:**
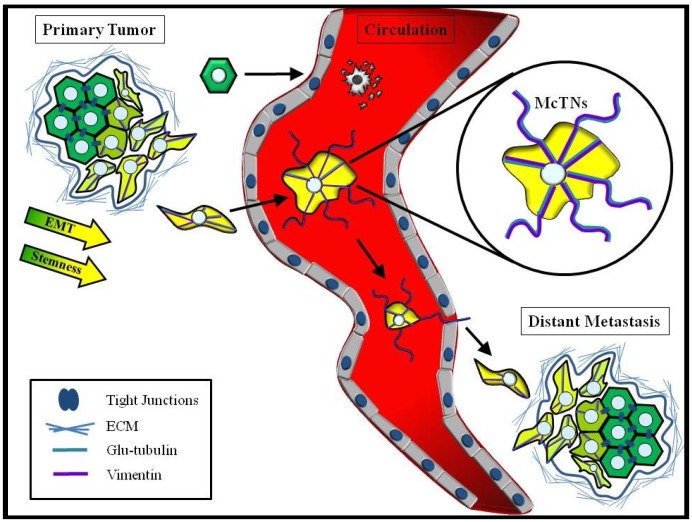
EMT and Stemness promote cytoskeletal alterations that enhance CTC reattachment. EMT and cancer stem cell traits cooperate to promote successful metastasis. Breast cancer cells with EMT and cancer stem cell characteristics (yellow cells), have enhanced invasive and migratory potential that aid in their ability to enter circulation as CTCs. Activated anoikis-resistance pathways and a more deformable cytoskeleton allow the circulating CSCs (yellow cells) to survive in the bloodstream, while more epithelial-like tumor cells (green cells) die by anoikis or fragmentation in the capillaries. Upregulation of the intermediate filament vimentin (purple) and detyrosination of α-tubulin to generate Glu-tubulin (teal) at the invasive front of the primary tumor predispose CTCs to produce microtentacles (McTNs) when suspended in circulation. These McTNs penetrate between endothelial cells junctions to promote CTC reattachment at a distal site, where CSC characteristics promote outgrowth as a metastatic lesion.

In addition to detecting CTCs with CSC character, many breast CTCs display evidence of EMT. Immunostaining of breast cancer patient CTCs revealed EMT markers Twist and vimentin in 73% and 77%, respectively, of CTCs from patients with early disease, and in 100% of the CTCs from patients with metastatic disease [92]. Interestingly, these findings of high Twist and vimentin levels in CTCs from patients with metastatic breast cancer support the model in which EMT promotes vimentin and glu-tubulin enriched McTNs to enhance CTC reattachment (Figure 2) [66,67]. A recent study by Yu *et al.* used dual-colorimetric RNA-in situ hybridization (RNA-ISH) to assess breast tumor cells for EMT markers in circulation and at the primary tumor site and draining lymph nodes [93]. They report finding biphenotypic tumor cells expressing both epithelial and mesenchymal markers at the primary tumor and draining lymph nodes, supporting the idea that EMT can start at the primary tumor to help cells escape and enter circulation, notably finding that mesenchymal cells were enriched in CTCs. Serial monitoring of patients revealed that disease progression was associated with an increase in these mesenchymal CTCs. RNA sequencing of the CTCs indicated an increased expression of TGF-β pathway components and the FOXC1 transcription factor, both strongly associated with EMT [93]. In a clinical study of patients with metastatic breast cancer undergoing high-dose chemotherapy with autologous hematopoietic stem cell transplantation, patients whose CTCs overexpressed EMT transcription factors had an increased risk of disease relapse and shorter progression-free survival times [94]. These studies suggest that EMT-associated CTCs contribute to metastasis and poor prognosis.

Providing further support for the connection between EMT and CSCs, patient CTCs with CSC characteristics were also found to have EMT markers [95]. Patient CTC samples with high expression of EMT transcription factor TWIST1 had a higher percentage of CSCs, as determined by Aldefluor assay, and patients who had a complete response had a significantly lower percentage of CSCs [94]. Recently, Zhang *et al.* isolated CTCs from the peripheral blood of patients with invasive breast cancer by multiparametric fluorescence-activated cell sorting. Interestingly, Zhang *et al.* relied on cancer stem cell marker ALDH1 in their CTC isolation, further emphasizing the role of CSC character for metastasis. After culturing these isolated CTCs, they found that these CTCs had high levels of the other breast CSC markers, CD44^hi^/CD24^lo^, and high levels of mesenchymal marker vimentin [90]. 

While CellSearch remains the only U.S. Food and Drug Administration-approved test for CTC enumeration, this test relies on the EpCAM antibody to identify CTCs and thus is unable to capture CTCs that have become more mesenchymal than epithelial and have downregulated EpCAM expression. If EMT increases the chances of successful metastasis, then the CellSearch method may not be the best choice for metastasis studies, as it would miss a significant fraction of CTCs having undergone EMT. In a prospective study of HER2^+^ patients with metastatic breast cancer, Giordano *et al.* isolated CTCs from the peripheral bloodstream and found a heterogeneous CTC population, where some CTCs expressing an EMT or CSC-like phenotype did not express EpCAM and thus would be missed by CellSearch [96]. These findings highlight the need to expand CTC detection methods to include CTCs that have undergone an EMT.

## 6. Chemotherapeutics Targeting Primary Tumor Growth may have Unintended Consequences for CTCs

Current clinical trials for cancer chemotherapeutics assess the ability to shrink existing tumors, rather than prevent metastatic spread and outgrowth. This approach neglects the fact that the biology of metastasis may be very different than that of primary tumor development [97]. Indeed, chemotherapeutics may have very different effects on tumor cells in an already established lesion than on CTCs in circulation. Studies using suspended breast cancer cell lines to model CTCs show that treatment with Taxol, a commonly used antiproliferative chemotherapeutic, enhances the level of detyrosinated tubulin and formation of McTNs, as well as their ability to reattach from suspension [98]. In addition to their effects on the cytoskeleton of CTCs, chemotherapeutics may have unintended effects on the behavior of CTCs. Adriamycin treatment can induce EMT in a Twist-dependent manner in breast cancer cells, providing an unintended metastatic advantage [99]. Additionally, irradiation, a common treatment modality in breast cancer, can increase EMT and CSC characteristics [100]. Importantly, patients receiving neoadjuvant therapy were more likely to express EMT transcription factors in their CTCs [101]. Given the connection between EMT and CSCs, and the therapeutic resistance associated with CSCs, this finding suggests that either EMT-like CTCs are resistant to neoadjuvant therapy, or that the stress of neoadjuvant therapy could trigger CTCs to undergo an EMT [101]. Taken together, these studies suggest that the effects of therapies on CTCs need to be considered when designing treatment plans with the aim of treating or preventing metastasis. A compelling recent study shows that when CTCs increase during neoadjuvant chemotherapy, breast cancer patients have a 25-fold higher risk of relapse after seven years, emphasizing the importance of understanding how existing cancer therapies affect CTC metastatic potential [102].

## 7. Conclusions

In order to enter the circulatory system, breast cancer cells must undergo extensive cytoskeletal alterations**.** The EMT program has been widely studied as a mechanism that enhances cancer cell motility and escape from the primary tumor, but recent studies using developing technology to isolate CTCs suggest that the EMT program provides additional advantages to cancer cells in the very different microenvironment of the circulatory system. The EMT program can be particularly advantageous for breast CTCs since it appears to increase invasiveness to aid in the generation of CTCs, confers resistance to anoikis once the cells are in circulation, and promotes McTN formation and CSC character, giving the CTCs a metastatic advantage in exiting the bloodstream and surviving to emerge later as dangerous metastatic lesions.
